# The effect of parenting style on aggressive behavior in adolescents: the chain mediating roles of self-control and attachment

**DOI:** 10.3389/fpsyt.2026.1741189

**Published:** 2026-03-11

**Authors:** Jin Xie, Ying Zou, Yang Peng, Can Zhou, Qingtao Dong, Zhendong Yao

**Affiliations:** 1 Mental Health Service Center, Huanghuai University, Zhumadian, China; 2 Counseling Center, Hunan Non-ferrous Metals Vocational And Technical College, Zhuzhou, China; 3 Psychological Counseling Center, Guangdong Polytechnic of Industry and Commerce, Guangzhou, China; 4 School of Art and Design, Huanghuai University, Zhumadian, China; 5 School of Educational Science, Hunan University of Arts and Science, Changde, China

**Keywords:** adolescent, aggressive behavior, attachment, parenting style, self-control

## Abstract

**Introduction:**

We investigated the mediating effects of self-control and attachment in the relationship between parenting style and aggressive behavior among Chinese adolescents.

**Methods:**

A total of 617 adolescents from Jiangsu, Henan and Hunan provinces completed Parenting Style Scale、 Aggressive Behavior Scale、 Self-Control Scale and Attachment Sale.

**Results:**

The results were follows: (1) There were significant correlations between parenting style, aggressive behavior, self-control, and attachment. (2) Both self-control and attachment mediated the relationship between positive parenting style and aggressive behavior. (3) Self-control and attachment played a significant chain mediating role in the relationship between positive parenting style and aggressive behavior.

**Discussion:**

Thus both parents’ positive and negative parenting styles can directly associate adolescents’ aggressive behavior. In addition, parents’ positive parenting style can also indirectly associate adolescents’ aggressive behavior through the separate mediating and the chain mediating roles of self-control and attachment.

## 1 Introduction

Aggressive behavior refers to behavior reactions or psychological tendencies that are intentional to cause harm to others (1). For adolescents, aggressive behavior is easily rejected by their peers, which associate difficulties in interpersonal communication and having negative influence on the acquisition of skills and learning (2). Aggressor can associate intense resentment and rebelliousness in adolescents. (3). At present, there are growing body of studies on the causes of adolescents’ aggressive behavior, but lack of discussion on the comprehensive mechanism of adolescents’ aggressive behavior from individual perspectives such as self-control and attachment (4). In view of the adverse effects of aggressive behavior on the physical and mental health of adolescents, it was of great practical significance to explore the formation mechanism of adolescents’ aggressive behavior for reducing aggressive behavior.

The formation mechanism of aggressive behavior is often attributed to situational factors, according to the General Aggression Model proposed by Anderson and Bushman (1), especially the parenting style in the family environment has an important influence on the formation of adolescents’ aggressive behavior (5). Research has classified parenting styles into three main types: authoritative, authoritarian, and permissive (6). Studies have shown that adolescents with authoritative parenting style were less likely to engage in aggressive behavior (7, 8), however, adolescents with autocratic parenting style tended to have more aggressive behaviors (9). In addition, adolescents with indulgent parenting style also tended to be more aggressive (10). A study suggested that aggression in adolescents should be studied by examining the parenting styles that adolescents experienced. Parenting styles have been categorized into rejection, over-protection, and emotional warmth, with the former two being negative and the latter being positive (11). A study have shown that adolescents who experience positive parenting styles are less likely to engage in aggressive behavior, whereas those exposed to negative parenting styles are more likely to exhibit aggressive behavior (12). Therefore, parenting style has a direct impact on adolescents’ aggressive behavior. However, the specific mechanism of how parenting style associate adolescents’ aggressive behavior is still unclear. This study will further explore the mechanism of parenting style on adolescents’ aggressive behavior.

As for the formation mechanism of aggressive behavior, the General Aggression Model maintain that an important factor leading to the emergence of aggressive behavior is the situational factor (1). Situational factors mainly come from the environmental stimuli around adolescents, especially the parenting style in the family environment has an important influence on the formation of adolescents’ aggressive behavior (5). Parenting style refer to the complex of parents’ rearing attitude, rearing method, rearing behavior and the emotional expression conveyed by parents’ behavioral responses in daily life, which are characterized by relative stability (13). Previous studies have divided the parenting styles into three types: authoritative, autocratic and indulgent (6). The authoritative parenting style is generally characterized by support, understanding, guidance and behavioral supervision. Autocratic parenting style has features such as control, beating and scolding, punishment, etc. The indulgent parenting style had the characteristics of indulgence, laissez-faire, tolerance and non-restraint. These three parenting styles have different influences on adolescents’ aggressive behavior. Studies have shown that adolescents with authoritative parenting style were less likely to engage in aggressive behavior (7, 8), however, adolescents with autocratic parenting style tended to have more aggressive behavior (9). In addition, adolescents with indulgent parenting style also tended to be more aggressive (10). Therefore, parenting style has a direct impact on adolescents’ aggressive behavior. However, the specific mechanism of how parenting style associate adolescents’ aggressive behavior is still unclear. A study suggests that aggression in adolescents should be studied by examining the parenting styles that adolescents experienced. The research divides parenting styles into three types: rejection, over-protection, and emotional warmth. The former two are negative parenting styles, and the latter is positive parenting style (11). According to this classification, a study point out that adolescents influenced by positive parenting style have less aggressive behavior, while adolescents influenced by negative parenting style have more aggressive behavior (12). Previous studies have also shown a negative correlation between aggressive behavior and perceived positive parenting style in adolescents (14). The dichotomy is rooted in a fundamental distinction between parenting behaviors focused on support, guidance, and reinforcement (positive) and those focused on punishment, control, or withdrawal of support (negative) (15). This binary does not claim parenting is strictly one or the other (most parents use a mix), but rather isolates two opposing core orientations that simplify the complexity of real-world parenting interactions. Under the premise of maintaining theoretical foundations, this study adopts the positive/negative dichotomy, which not only enhances the robustness of measurement and modeling but also aligns with intervention and cross-cultural tools, consistent with recent research findings on the direction of effects (16). Based on this classification, this study put forward Hypothesis 1: Positive parenting style was negatively correlated with adolescents’ aggressive behavior; self-control and attachment were both significantly negatively correlated with aggressive behavior.

Self-control refers to the ability of individuals to monitor, restrain, persist and adjust their own behaviors, emotions and expectations in order to achieve specific goals (17), which will associate adolescents’ mental health (18). According to self-control theory (19), compared with individuals with high self-control ability, individuals with low self-control are more prone to have aggressive behavior (20, 21). Studies have found that self-control ability has a significant negative correlation with aggressive behavior (22). Some studies have also pointed out that adolescents with good self-control ability are better able to cope with stress, less likely to be irritated and less aggressive (23), while adolescents with low self-control have more interpersonal conflicts and aggressive behaviors (24). Positive parenting styles, which are crucial for the development of adolescent self-control (25), help adolescents internalize external norms, thereby facilitating the development of effective self-control (26). Conversely, negative parenting styles such as parental punishment and neglect may associate low self-control of adolescents, and high self-control is likely to reduce adolescents’ aggressive behavior (27). Based on this, we proposed Hypothesis 2: parenting style associated adolescents’ aggressive behavior through the mediating effect of self-control.

Previous studies have shown that once the attachment relationship established, it would remain relatively stable and associate the temperament, personality, emotion and social behaviors of adolescents (28). According to attachment theory, the inter-generational transmission of attachment is influenced by internal working model formed in early life. The model encompass an individual’s perceptions of self and others, as well as the behavioral patterns from early parent-child interactions. Once this pattern was internalized into the individual consciousness, it will be relatively stable, and play a pivotal role in the individual’s subconscious, and form their own way of expressing intimacy (29). Studies have shown a significant link between early attachment experiences and aggressive behavior in adolescents, adolescents with secure attachment tend to exhibit less aggression compared to those with insecure attachment (30, 31). Some studies have also pointed out that the insecure parent-child attachment associate more negative cognition, less acceptance of others, poor interpersonal relationship and more aggressive behaviors in the left-behind adolescents (32, 33). Adolescents who experience punitive parenting are more likely to develop insecure attachment. As they mature, they tend to incorporate the negative parenting they have experienced into their social interactions, which can impair their interpersonal skills, foster rebellious or avoidant personality traits, and increase the likelihood of engaging in aggressive behavior (34, 35). Hence, this research put forward Hypothesis 3: Parenting style associated adolescents’ aggressive behavior through the mediating effect of attachment.

Previous research have primarily investigated the relationships between two or three of the following factors: parenting styles, aggressive behavior, self-control, and attachment. However, studies examining the relationship between all four factors are scarce. Parenting styles not only influence adolescent aggressive behavior, but this relationship is also mediated by the quality of attachment and the adolescent’s level of self-control. For example, even with a democratic and permissive parenting style, the likelihood of aggressive behavior in adolescents may not be reduced; the quality of the attachment between the adolescent and their parents is also a critical factor to consider (36). Therefore, we proposed Hypothesis 4: Parenting style associated adolescent aggression through the chain mediating effect of self-control and attachment.

**Figure 1:**
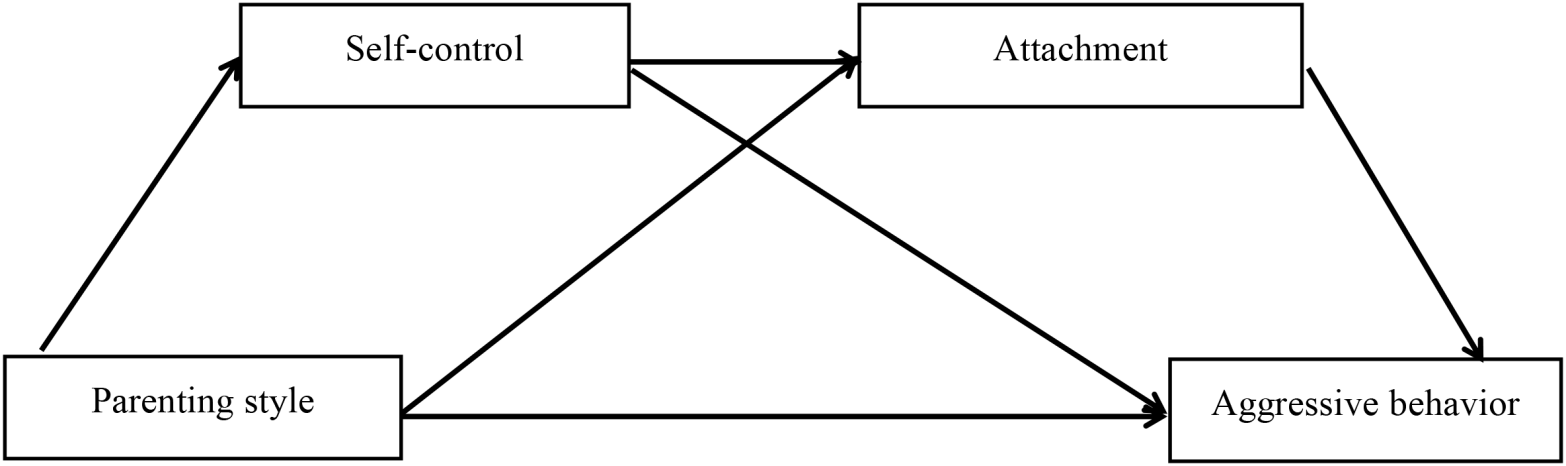
A chain mediation model of self-control and attachment in the influence of parenting styles on adolescent aggressive behavior.

The detailed hypothetical model was shown in **Figure 1**. Self-control is a core regulatory skill necessary for navigating social interactions (37). The adolescents must be able to modulate impulses (e.g., crying, reaching) to engage in the synchronized “serve-and-return” interactions that build secure attachment. In this view, basic self-regulation is a prerequisite for optimal attachment development. In proposing this model, we assume a pathway where early-emerging self-control capacities facilitate the development of secure attachment.

## 2 Methods

### 2.1 Participants

A total of 660 questionnaires were distributed from three schools in Jiangxi, Hunan and Henan provinces by convenient sampling method. Under the premise of obtaining the consent of the subjects, the group test was conducted in class, the questionnaires were collected on the spot. After removing 43 invalid questionnaires, 617 were obtained, with a ratio of 93.48%. The sample included 327 boys and 290 girls, with 147 students in the sixth grade of primary school, 256 students in the first grade of middle school and 214 students in the second grade of middle school. The mean age of all subjects was 13.77 years, with a standard deviation of 1.78 years.

### 2.2 Measures

#### 2.2.1 Parenting style scale

The Simple Parenting Style Scale which was compiled by Arrindell (11) and revised by Chinese researchers Jiang (38) was adopted to measure the parenting style of adolescents. The scale included three dimensions of rejection, emotional warmth and over-protection, with a total of 21 items (e.g., “Allow adolescents to express their views freely”), which were rated on a 5-point Likert scale from 1 (never) to 5 (always). Based on previous studies (39), this study explored the parenting style from two aspects: positive parenting style (emotional warmth) and negative parenting style (rejection and over-protection). The total scoring method was based on two dimensions: positive parenting style and negative parenting style. The higher the score, the more inclined to adopt this parenting style. The Cronbach’s α coefficients of positive parenting style and negative parenting style were 0.89 and 0.88, respectively. The results of confirmatory factor analysis showed that the structure validity of the scale was good: χ^2^/df =3.98, CFI = 0.89, NFI = 0.94, GFI = 0.84, RMSEA = 0.042.

#### 2.2.2 Aggression scale

The Aggression Scale which was compiled by Buss and Perry (40) contained four dimensions: physical aggression, verbal aggression, anger and hostility, with a total of 29 items (e.g. “I threatened people I knew.”). Adolescents rated each item on a 5-point scale (1 = complete disagreement, 5 = complete agreement). The total scores of all the items were added together. The higher the score, the stronger the aggression. This scale has shown good reliability and validity in previous research (41). In this study, the Cronbach’s α coefficient of internal consistency of the scale was 0. 84, and the Cronbach’s α coefficients of physical aggression, verbal aggression, anger and hostility were 0.81, 0.82, 0.83 and 0.85, respectively. Confirmatory factor analysis showed that the construct validity of the scale was good: χ^2^/df = 3.62, CFI = 0.94, NFI = 0.93, GFI = 0.96, RMSEA = 0.03.

#### 2.2.3 Self-control scale

The Self-control Scale which was compiled Tangney et al. (42) and revised by Tan et al. (43) was used to measure the self-control ability of adolescents. The scale had 19 items in total, including two dimensions of self-discipline and impulse control. Each item (e.g. “When I face temptation, I have a great chance to resist it.”) was rated on a 5-point Likert scale, from “1 = completely disagree” to “5 = completely agree”. The total scores of all the items were added together. The higher the score, the stronger the individual’s self-control ability. In this study, the Cronbach’s α coefficient of internal consistency of the scale was 0. 83, and Cronbach’s α coefficient of internal consistency of self-discipline and impulse control dimensions was 0.80 and 0.84, respectively. Confirmatory factor analysis showed that the construct validity of the scale was good: χ^2/^df = 2. 98, CFI = 0.93, NFI = 0.92, GFI = 0.95, RMSEA = 0.031.

#### 2.2.4 Attachment scale

In this study, the parental peer attachment scale which was compiled by Armsden & Greenberg (44) was used to measure the degree of parent-child attachment between adolescents and their parents. The scale included 3 dimensions (total scores of trust, communication, and alienation), with a total of 25 items. A 5-point Likert scale was used in the questionnaire, with 1 indicating “never” and 5 indicating “always”. The total score was obtained by adding up the original scores for trust and communication, and then subtracting the score for alienation (after reverse-scoring relevant items), which was used to evaluate the attachment quality of adolescents. In this study, the Cronbach’s α coefficient of internal consistency of the scale was 0. 86, and the Cronbach’s α coefficient of internal consistency of the total score of trust, communication, attachment and were 0. 83, 0. 85, and 0. 86, respectively. Confirmatory factor analysis showed that the construct validity of the scale was good: χ2/df = 3. 42, CFI = 0. 95, NFI = 0. 94, GFI = 0. 96, RMSEA = 0.04.

### 2.3 Procedures and data analysis

In this study, a group test was conducted by a professionally trained assistants. The scales were distributed and instructions were read to the subjects. The subjects were allowed to answer under the premise of understanding the questionnaire task and the principle of anonymity. The scales were collected on the spot after all the participants completed. In this study, SPSS 22.0 was adopted to analysis the data, and common method bias test、descriptive statistics and correlation analysis were carried out. In addition, AMOS23.0 was used to construct the structural equation model.

## 3 Results

### 3.1 Common method bias test

During the data processing phase, the Harman single-factor test was employed to assess common method bias. The findings revealed that the first factor accounted for only 27.82% of the variance, falling short of the critical value of 40%, indicating that common method bias is not statistically significant (45).

### 3.2 Descriptive statistics and correlation analysis of each variable

Statistical analysis (see **Table 1**) revealed that positive parenting styles were significantly positively correlated with self-control and attachment, while significantly negatively correlated with aggressive behavior. Conversely, negative parenting styles were significantly negatively correlated with self-control and attachment, and significantly positively correlated with aggressive behavior. Additionally, there was a significant positive correlation between self-control and attachment, with both showing significant negative correlations with aggressive behavior. Finally, a significant negative correlation was found between attachment and aggressive behavior.

**Table 1:** Descriptive Statistics and Correlation Analysis Results for Each Variable.

Variables	*M*	*SD*	1	2	3	4	5
1PP	39.18	6.26	–				
2NP	52.32	9.59	-0.30**	–			
3SC	61.96	11.00	0.22**	-0.15**	–		
4AT	29.67	3.32	0.10**	-0.13**	0.43**	–	
5AB	32.40	3.47	-0.37**	0.03**	-0.15**	-0.44**	–

**p < 0.01. The data of each variable have been standardized, the same below.

PP, Positive Parenting; NP, Negative Parenting; SC, Self-Control; AT, Attachment; AB, Aggressive Behavior (abbreviations will be used henceforth).

### 3.3 Test of mediating effect

**Figure 2:**
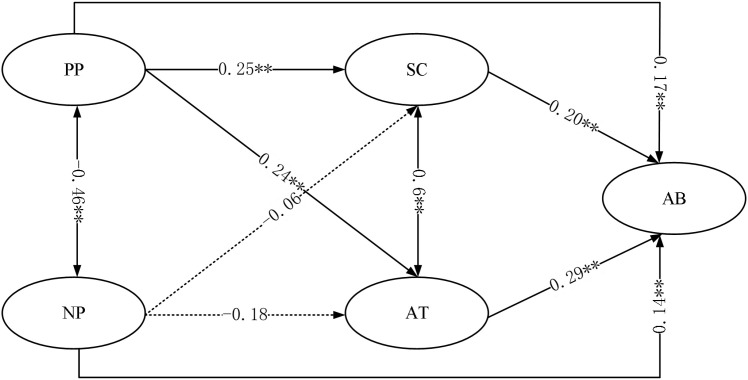
The mediation model.

Based on the hypothesis model and the analysis of the relationship between each variable, a chain mediation structural equation model with latent variables was established to investigate the mediating effect among parenting style, self-control, attachment, and aggressive behavior. The results showed that the model’s fit indices were as follows: χ^2/df = 4.33, NFI = 0.95, CFI = 0.94, IFI = 0.92, RMSEA = 0.06, indicating a good model fit (see **Figure 2**). Both positive parenting style and negative parenting style had a significant effect on aggressive behavior (β = 0.49, *p < 0.001*; β = 0. 48, *p < 0. 001*).

The results showed that positive parenting style had significant direct effects on aggressive behavior (*β* = 0.17, *p* < *0.01*), self-control (β= 0.25, *p < 0.01*) and attachment (*β* = 0.24, *p < 0.01*). Negative parenting style had a significant direct effect on aggressive behavior (*β* = 0. 14, *p < 0.01*), but the direct effects on self-control (*β* = -0. 06, *p > 0.05*) and attachment (*β* = -0. 18, *p > 0.05*) were not significant. ** *p* < *0.01*.

### 3.4 Significant test and effect analysis of the mediating effects of self-control and attachment

The mediating effects of the model were tested using the bias-corrected percentile Bootstrap method with 5000 samples. The results showed that positive parenting style had a significant indirect effect on aggressive behavior (β = 0.33, SE = 0.05, 95% confidence interval [0.25, 0.37]), and negative parenting style also had a significant indirect effect on aggressive behavior (β = -0.05, SE = 0.01, 95% confidence interval [-0.09, -0.02]). The specific indirect effects and their 95% confidence intervals estimated by the Bootstrap method are presented in **Table 2**.

**Table 2:** Mediating Effects and 95% Confidence Intervals Estimated by Bootstrap Methods for Each Indirect Effect.

Pathways	Standardized indirect effect estimates	95% confidence interval	
		Lower	Upper
PP—AB	0.20	0.14	0.26
PP—SC—AB	0.01	0.01	0.02
PP—AT—AB	0.06	0.05	0.10
PP—SE—AT—AB	0.01	0.01	0.02
NP—AB	-0.04	-0.05	-0.01
NP—SC—AB	0.08	-0.05	-0.04
NP—AT—AB	0.06	0.01	0.02
NP—SC—AT—AB	0.03	-0.03	-0.01

## 4 Discussion

The present model—linking parenting → self-control → attachment → aggression—is consistent with: (i) longitudinal/meta-analytic evidence that parenting practices are associated with adolescents’ self-control (26); (ii) transactional findings that youths’ regulatory capacities help sustain higher-quality relationships (felt security/less conflict) (46); and (iii) robust literature connecting attachment security and self-control with lower aggression. Together, these strands support a cascade in which supportive parenting fosters adolescent self-control, which facilitates more secure attachment, which in turn reduces aggression (47, 48).

### 4.1 The correlation between parenting style, self-control, attachment, and aggressive behavior

The results of the study show that positive parenting style is significantly negatively correlated with aggressive behavior; both self-control and attachment are significantly negatively correlated with aggressive behavior, which verify all the contents of Hypothesis 1. Under the influence of positive parenting style, adolescents can receive more warmth and support, thus facing setbacks with a more positive attitude. Positive parenting style is conducive to adolescents forming a correct understanding of objective matters, and at the same time, they have good self-monitoring and self-restraint on their negative behaviors, which can effectively resist negative emotions such as anxiety, and avoid aggressive behaviors, which is similar to previous research results (49). In this study, it is found that self-control is negatively correlated with aggressive behavior, which is consistent with previous research results (50). Adolescents with high self-control tend to have better emotional regulation abilities, who can deal with the pressure in daily life and have stronger adaptability (51). On the contrary, adolescents with low self-control prefer immediate gratification, and if their desires are not met promptly, it may associate aggressive behavior. In this study, it is found that attachment is negatively correlated with aggressive behavior, which is consistent with previous research results (33). It is suggested that the safer the attachment relationship, the less aggressive behavior adolescents exhibit. Secure attachment promotes internal feelings of safety and trust, which enable adolescents to regulate negative emotions, develop empathy, and rely on constructive coping strategies rather than aggression when faced with conflict. Besides, the results suggests that negative parenting style foster aggression partly by creating a relational context of insecurity and distrust. This may lead adolescents to perceive the world as hostile and to adopt pre-emptive aggressive strategies in social interactions. Negative parenting style also directly impedes the acquisition of self-regulatory skills, perhaps by failing to model or reinforce calm problem-solving, or by escalating emotional arousal that overwhelms the adolescent’s regulatory capacity (52).

### 4.2 The mediating effect of self-control

Individuals with high self-control are able to effectively monitor, regulate, and inhibit their emotions, cognition, and behaviors. They can think about problems from different angles, assess the situation, and solve problems in a more appropriate manner, thus exhibiting fewer aggressive behaviors. This study find that adolescent self-control mediates the relationship between positive parenting style and aggressive behavior, which verifies the contents of Hypothesis 2. This suggests that positive parenting style helps to reduce aggressive behavior in adolescents, and high self-control enables adolescents to focus their efforts on achieving goals (53). When adolescents’ needs cannot be met immediately, they may generate motivations and behaviors opposite to their parents, leading to aggressive behavior towards the outside world (54).

The mediating role of self-control may stem from its fundamental function of self-regulation. An active parenting style provides adolescents with a structured environment for learning behavioral rules and emotional management, which directly contributes to the development of self-control skills (55). And the development of good self-control skills enables teenagers to manage negative emotions and impulsive reactions in interpersonal conflicts more effectively, which may promote more harmonious and reliable parent-child interactions. This creates the conditions for maintaining a secure attachment relationship (56). This might partly explain why self-control plays a mediating role before attachment: it is more like an immediate and dynamic “regulator” that directly affects the experience and expression of the attachment relationship in daily interactions.

### 4.3 The mediating effect of attachment

The results of the mediating effect test in this study show that attachment is an important mediating variable between parenting style and aggressive behavior, so Hypothesis 3 is verified. Attachment style is an important factor that determine adolescents’ interpersonal behavior. To a certain extent, it is generally believed that attachment style associated social interaction behaviors related to interpersonal care (57). Previous studies have shown that individuals with secure attachment have better performance in interpersonal behaviors, and they were more harmonious with others and more satisfied with interpersonal relationships than individuals with insecure attachment (58). Based on Bowlby’s attachment theory, infants and young children can judge their own popularity and others’ trustworthiness according to their caregivers’ timely, sensitive and consistent responses, and they gradually internalized the information in close contact with their main caregivers, eventually they formed an internal working model of attachment, and at the same time, they have obtained basic trust and established a prototype of interpersonal communication (59). Previous studies have shown that when parents held negative parenting styles, adolescents were more likely to have problems in attachment, which associate aggressive behavior (60).

### 4.4 The chain mediating effects of self-control and attachment

The results find that positive parenting style can influence aggressive behavior through a chain mediating effect of adolescents’ self-control and attachment, thus Hypothesis 4 is verified. This may be because parents’ positive parenting style conveys more warmth and clearer behavioral standards, and these parent-child interactions can help adolescents internalize external rules and expectations, promoting the development of self-control in adolescents, which in turn help adolescents reduce aggressive behavior. In contrast, under negative parenting styles, interactions are less frequent and lower quality, behavioral standards are vague, and the family atmosphere is more stressful, which is unfavorable for the development of self-control in adolescents (26). Most importantly, the significant chain pathway clarifies a key mechanism: negative parenting style first erodes the secure attachment bond. This insecure foundation fails to provide the emotional safety necessary for practicing and internalizing self-control. Consequently, the adolescents enter peer conflicts with both heightened relational insecurity and diminished regulatory tools, making aggressive responses more likely. Additionally, self-control is an important prerequisite for individuals to achieve self-regulation, and individuals with higher self-control are more inclined to allocate more psychological resources for their goals (61). According to the dual-component theory of self-control (62), proactive self-control enables adolescents to take continuous actions in the process of pursuing goals, exhibit more positive behaviors, and actively seek problem-solving, which is of great significance for reducing aggressive behaviors. Risky attachment can associate more negative cognition in adolescents, less acceptance of others, poor interpersonal relationships, and consequently more aggressive behavior. Parenting style exerts a subtle influence on adolescents. Through positive parenting style, a secure attachment relationship can be established, which allow adolescents to feel safe and effectively resist the interference of negative emotions, establish good interpersonal relationships, and thus reduce aggressive behavior (33).

The chain mediation model in this study indicates that parenting styles influence adolescents’ aggressive behavior through their effects on attachment and self-control. Furthermore, this finding can be logically and coherently explained within a series of developmental psychology theories. According to the tripartite model of familial influence (63), warm and supportive parenting provides adolescent with a low-stress and highly supportive environment, which facilitates the development of executive functions and thereby lays the foundation for self-control. Conversely, harsh or neglectful parenting tends to evoke chronic stress in adolescent, depleting the cognitive resources necessary for effective self-regulation. Simultaneously, according to Social Imitation Learning Theory (64), adolescents internalize behavioral norms and learn to practice delayed gratification and impulse inhibition through consistent parental rule-setting and feedback. Furthermore, according to Attachment Theory (59), adolescents with secure attachment internalize the parent-child relationship as a reliable source of support. When confronted with interpersonal conflicts, their secure internal working models predispose them to make non-hostile attributions and to believe that problems can be resolved through communication rather than aggression, thereby significantly reducing the likelihood of aggressive behavior.

## 5 Limitations and future directions

This study has made preliminary findings, but there are still some issues that need to be further improved in future research: First, cross-sectional investigations were adopted in this study to examine the self-control and attachment of adolescents in the relationship between the parenting style and aggressive behavior, but there was no study on the development process between them from the perspective of dynamic development. In subsequent research, longitudinal tracking research methods can be conducted to investigate the relationship between adolescents’ self-control, attachment, parenting style, and aggressive behavior. Second, in this study, self-report questionnaires are used to collect data, therefore, a “social approval effects” may exist, which associate the expectation effect when adolescents filled in the questionnaires. In the future research, the “double-blind design” experimental methods can be considered to avoid the expectation effect of the adolescents. Third, this study does not discuss the role of parenting styles of father and mother separately, which can be further discussed from this perspective in the future. Besides, the generalizability of the study is limited due to the use of convenience sampling in this research. The limitations of relying solely on Harman’s single-factor test for common method bias, such as low detection power and the potential to provide a false sense of security, which may cause errors conclusions in this study. To address these issues, future research designs should consider separating data sources in advance and employ statistical control methods based on the confirmatory factor analysis (CFA) framework during data analysis.

## 6 Implications and significance of the research

According to the results of this study, the parenting style exerts an extremely significant influence on the aggression of adolescents through the chain-mediated effect of self-control and attachment. This provides profound inspiration for reducing adolescents’ aggressive behavior. First of all, in the process of child-rearing, parents should adopt scientific parenting styles to provide a conducive family environment for their adolescents’ healthy development. They ought to engage in frequent emotional communication, learn to listen attentively to their adolescents’ perspectives, and offer appropriate guidance to facilitate the development of sound cognitive patterns. Additionally, schools should provide adolescents with activities such as group psychological counseling and summer camps to increase their opportunities for engagement, enhance their sense of community, belonging, and security, and encourage them to adopt positive coping strategies when facing negative events or emotions, thereby avoiding the use of extreme responses such as aggression. Thirdly, schools should offer more mental health education courses to guide adolescents in developing realistic self-perception and self-evaluation, enhance their volitional qualities and self-regulatory capacity, prevent the emergence of aggressive behaviors, and thereby facilitate their positive psychosocial development.

## Data Availability

The datasets presented in this article are not readily available due to privacy or ethical restrictions. Requests to access the datasets should be directed to the corresponding author/s.
